# Spatial and risk factor analyses of vector-borne pathogens among shelter dogs in the Eastern United States

**DOI:** 10.1186/s13071-023-05813-1

**Published:** 2023-06-10

**Authors:** Corinna M. Hazelrig, Jenna R. Gettings, Christopher A. Cleveland, Andrea Varela-Stokes, Ania A. Majewska, Kris Hubbard, K. Wade Burton, Michael J. Yabsley

**Affiliations:** 1 Southeastern Cooperative Wildlife Disease Study, Department of Population Health, College of Veterinary Medicine, University of Georgia, Athens, GA USA; 2 Warnell School of Forestry and Natural Resources, University of Georgia, Athens, GA USA; 3 Department of Comparative Biomedical Sciences, College of Veterinary Medicine, Mississippi State University, Mississippi State, MS USA; 4 Department of Physiology and Pharmacology, College of Veterinary Medicine, University of Georgia, Athens, GA USA; 5 Center for Ecology of Infectious Diseases, University of Georgia, Athens, GA USA; 6 IDEXX Laboratories, One IDEXX Drive, Westbrook, ME USA; 7 Present Address: Department of Comparative Pathobiology, Cummings School of Veterinary Medicine, Tufts University, North Grafton, MA USA; 8 Present Address: West Asheville Family Vet, Asheville, NC USA

**Keywords:** Co-infections, Heartworm, Tick-borne disease, Vector-borne disease

## Abstract

**Background:**

Vector-borne infections pose significant health risks to humans, domestic animals, and wildlife. Domestic dogs (*Canis lupus familiaris*) in the United States may be infected with and serve as sentinel hosts for several zoonotic vector-borne pathogens. In this study, we analyzed the geographical distribution, risk factors, and co-infections associated with infection with *Ehrlichia* spp., *Anaplasma* spp., *Borrelia burgdorferi*, and *Dirofilaria immitis* in shelter dogs in the Eastern United States.

**Methods:**

From 2016 to 2020, blood samples from 3750 shelter dogs from 19 states were examined with IDEXX SNAP^®^ 4Dx^®^ Plus tests to determine the seroprevalence of infection with tick-borne pathogens and infection with *D. immitis*. We assessed the impact of factors including age, sex, intact status, breed group, and location on infection using logistic regression.

**Results:**

The overall seroprevalence of *D. immitis* was 11.2% (*n* = 419/3750), the seroprevalence of *Anaplasma* spp. was 2.4% (*n* = 90/3750), the seroprevalence of *Ehrlichia* spp. was 8.0% (*n* = 299/3750), and the seroprevalence of *B. burgdorferi* was 8.9% (*n* = 332/3750). Regional variation in seroprevalence was noted: *D. immitis* (17.4%, *n* = 355/2036) and *Ehrlichia* spp. (10.7%, *n* = 217/2036) were highest in the Southeast while seroprevalence for *B. burgdorferi* (19.3%, *n* = 143/740) and *Anaplasma* spp. (5.7%, *n* = 42/740) were highest in the Northeast. Overall, 4.8% (*n* = 179/3750) of dogs had co-infections, the most common of which were *D. immitis*/*Ehrlichia* spp. (1.6%, *n* = 59/3750), *B. burgdorferi/Anaplasma* spp. (1.5%, *n* = 55/3750), and *B. burgdorferi*/*Ehrlichia* spp. (1.2%, *n* = 46/3750). Risk factors significantly influenced infection across the evaluated pathogens were location and breed group. All evaluated risk factors were significant for the seroprevalence of *D. immitis* antigens.

**Conclusions:**

Our results demonstrate a regionally variable risk of infection with vector-borne pathogens in shelter dogs throughout the Eastern United States, likely due to varying distributions of vectors. However, as many vectors are undergoing range expansions or other changes in distribution associated with climate and landscape change, continued vector-borne pathogen surveillance is important for maintaining reliable risk assessment.

**Graphical Abstract:**

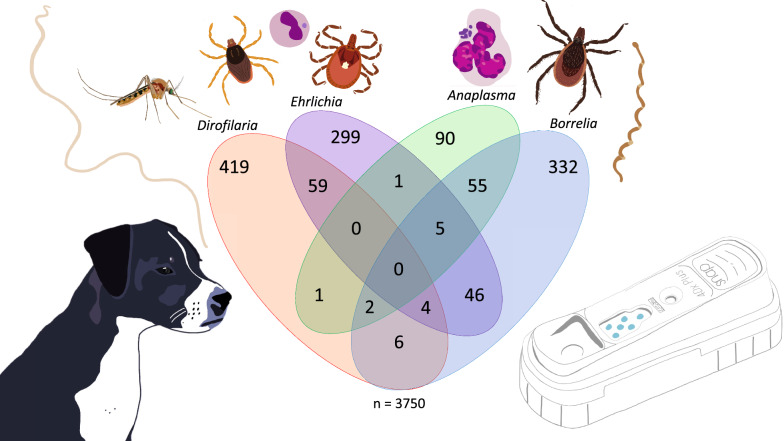

**Supplementary Information:**

The online version contains supplementary material available at 10.1186/s13071-023-05813-1.

## Background

Vector-borne infections pose significant health risks to humans, domestic species, and wildlife. Domestic dogs (*Canis lupus familiaris*) are susceptible to many vector-borne pathogens (VBP) of veterinary and public health concern [1–6], with *Dirofilaria immitis* (heartworm)*, Borrelia burgdorferi* (Lyme disease), *Anaplasma* spp. (anaplasmosis), and *Ehrlichia* spp. (ehrlichiosis) being some of the most studied pathogens [7, 8]. Importantly, several of these pathogens are significant pathogens of domestic dogs [9]. Finally, because several of these pathogens are zoonotic, dogs can serve as sentinel hosts. Using Lyme disease as an example, seroprevalence of *B. burgdorferi* infections in dogs is associated with counties where human infections occur [6, 10]. For these reasons, understanding the epidemiology of vector-borne infections in dogs is important for both human and veterinary health.

The origin and history of individual dogs is key to effective surveillance of canine VBP. Generally, the domestic dog population can be divided into groups such as owned dogs with veterinary care, owned dogs without veterinary care, stray dogs, and shelter dogs. The assumption is that many owned dogs under veterinary care would receive a combination of preventatives specific to VBP and intestinal parasites [9, 11, 12]. Dogs without veterinary care (owned or not) and those that enter shelters (generally > 50% of which are strays) are likely to be at a higher risk of infection with VBP through increased environmental exposure and/or lack of preventatives [7, 13, 14]. Other potential risk factors associated with an increased likelihood of VBP infection include age and body condition [15]. Older dogs will have a higher probability of being exposed during their lifetime, and dogs in poor body condition may be immunologically compromised, increasing infection risk.

Co-infections of vertebrate hosts are common and may complicate diagnosis and treatment or increase the risk of severe disease [4, 16–18]. In general, co-infections occur with pathogens with a common vector and/or overlapping geographical ranges [4, 7, 9, 11, 12]. For the pathogens of interest in our study, *D. immitis* is a mosquito-borne parasite, while the most common vector of *B. burgdorferi* and *Anaplasma phagocytophilum* is *Ixodes scapularis*, the most common vector of *Ehrlichia canis* is *Rhipicephalus sanguineus* and presumptively *Anaplasma platys*, and *Amblyomma americanum* is a common vector of *Ehrlichia chaffeensis* and *Ehrlichia ewingii* [7, 19].

The geographical distribution of tick species is changing, and thus the risk of co-infections is changing. This is reflected in the changes in the distribution and prevalence of canine VBP, highlighting the need for contemporary data [5, 8, 20–23]. In the Southeastern United States, co-infections between *D. immitis* and *Ehrlichia* spp. have been common while co-infections with *B. burgdorferi* and *A. phagocytophilum* are more common in the Upper Midwest and Northeast [1, 9, 11, 12]. The increasing detection of *A. americanum* in northeastern states could increase the risk of *Ehrlichia* infections in dogs in that region [24, 25]. Infection with many tick-borne pathogens causes similar clinical signs and presentations, but may require different treatments (e.g., *Babesia* spp.); thus, knowledge regarding the risk of co-infections is important. The purpose of this study is to evaluate the seroprevalence of select VBP (*D. immitis, Ehrlichia* spp., *Anaplasma* spp., and *B. burgdorferi*) in shelter dog populations in the Eastern United States and evaluate risk factors associated with seroprevalence and co-infections.

## Methods

### Sample collections

**Fig. 1:**
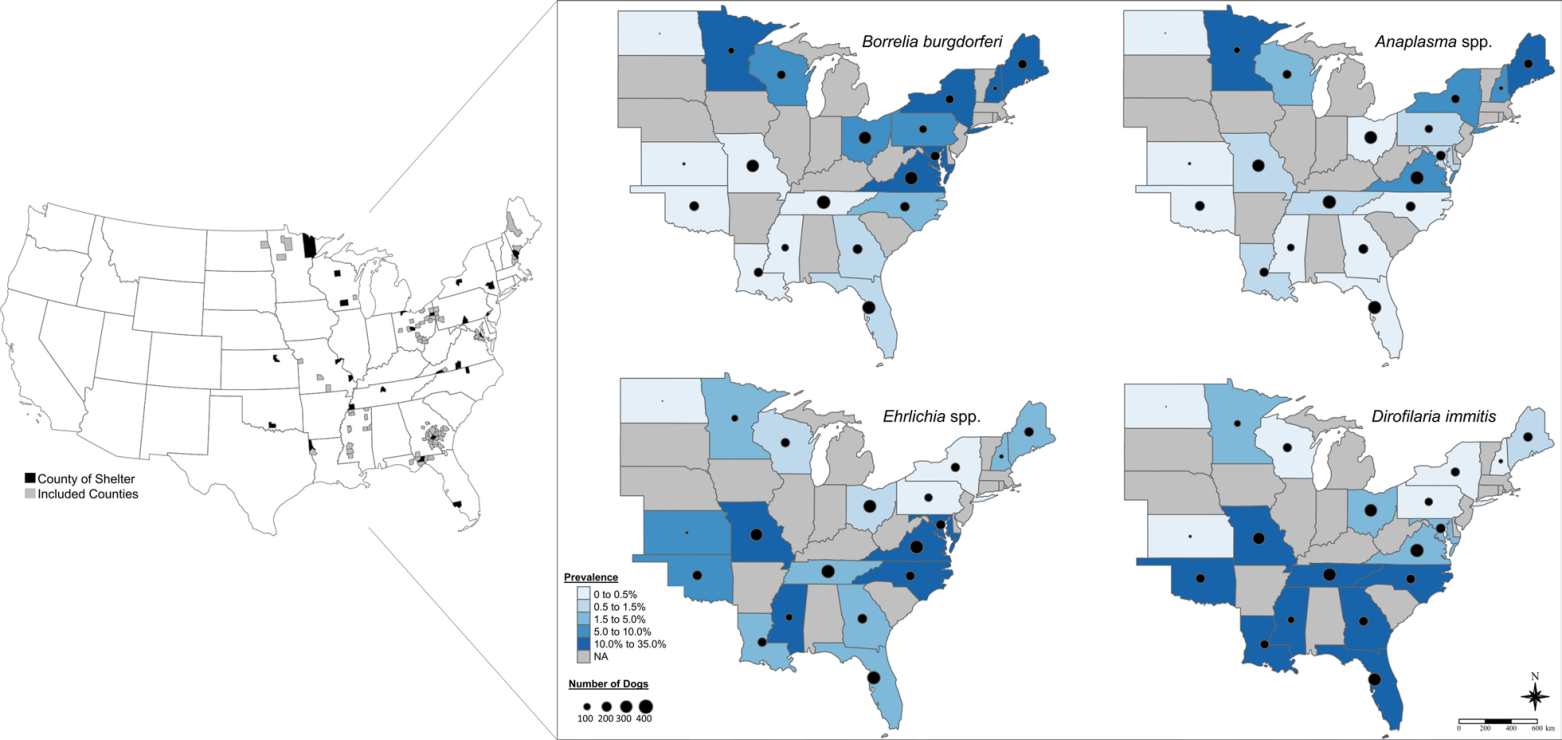
Map of the counties included in the study and seroprevalence maps of *Borrelia burgdorferi*, *Anaplasma* spp., *Ehrlichia* spp., and *Dirofilaria immitis.* For the county map, gray counties are where dogs originated from and black counties are where the shelters are located. For the seroprevalence map, the circles denote the approximate number of dogs tested in the state, and states in gray were not included in the study. The maps were created in R statistical software (R Core Team)

From 2016 to 2020, we recruited shelters in 19 states to participate in the study. Focal areas included 97 counties in the eastern half of the United States and were selected due to known geographical ranges of the focal pathogens (Fig. 1). Participating shelters were either asked to test dogs that met the inclusion criteria or provide data that was collected using the same methods and inclusion criteria. To be included, dogs had to be 6 months of age or older and had to originate from the county or neighboring counties around the shelter. Dogs that were too young or under bite quarantine were excluded. Dog blood samples were tested using SNAP^®^ 4Dx^®^ Plus tests which were provided by IDEXX Laboratories and shipped directly to the shelter or to the University of Georgia (UGA) for sample testing. We requested that shelters test between 50 and 200 dogs. Furthermore, SNAP^®^ 4Dx^®^ Plus data were provided by investigators from a related shelter study in Mississippi, which included data collected from June 2016 through February 2017 [15]. The approximate age, location, date the sample was collected, estimated breed and the corresponding American Kennel Club (AKC) breed group, sex, and intact status were collected for each dog.

### Data analysis

Variables assessed as risk factors included age (< 1 year old or ≥ 1 year old), location, breed group, intact status, and sex. Dogs were classified into AKC breed groups based on dominant breed features because many of the dogs were of mixed breed. Each pathogen and co-infection combination were evaluated using binomial generalized linear models (GLMs) to assess the relationship between the risk factor and the SNAP^®^ 4Dx^®^ Plus test outcome (positive, negative). Significant results from the GLMs were further analyzed through pairwise comparison to calculate the odds ratio (OR), confidence interval (CI), and *P*-value using the *lsmeans* package [26]. *P*-values were adjusted to correct for multiple comparisons using Tukey’s honestly significant difference test. All pathogens were assessed on an individual basis per pathogen and further analyzed through observed co-infection combinations, excluding dogs with no applicable values for the variable being analyzed. Statistical analyses were performed in R version 4.1.1 [27] and factors with *P* ≥ 0.05 were considered significant.

## Results

### Population data

The study included 3750 dogs from 19 states and 97 counties with dogs per county ranging from two to 226. The demographic data of the dogs in our study population can be found in Table 1. Most dogs were ≥ 1 year and intact; similar numbers of males and females were sampled (Table 1). The AKC breed group with the largest representation was the terrier breed group (*n* = 1185/3750, 31.6%) (Table 1). Nearly half (*n* = 2036/3750, 54.3%) of the dogs were from the Southeast with the remaining were from the Midwest (*n* = 974/3750, 26.0%) and Northeast (*n* = 740/3750, 19.7%) (Table 1).

**Table 1:** Demographic data and seroprevalence of infection with vector-borne pathogens among 3750 dogs from shelters in 19 states in the Eastern United States

	No. of samples	Number of dogs positive (%)			
		*Dirofilaria immitis*	*Anaplasma* spp.	*Ehrlichia* spp.	*Borrelia burgdorferi*
Total sample	3750	419 (11.2)	90 (2.4)	299 (8.0)	332 (8.9)
Age group					
< 1 year old	492	6 (1.2)	8 (1.6)	13 (2.6)	15 (3.0)
≥ 1 year old	3100	405 (13.1)	75 (2.4)	283 (9.1)	305 (9.8)
Unknown	158	8 (5.1)	7 (4.4)	3 (1.9)	12 (7.6)
Sex					
Female	1938	202 (10.4)	47 (2.4)	154 (7.9)	178 (9.2)
Male	1686	213 (12.6)	39 (2.3)	143 (8.5)	145 (8.6)
Unknown	126	4 (3.2)	4 (3.2)	2 (1.6)	9 (7.1)
Intact status					
Intact	2163	312 (14.4)	47 (2.2)	191 (8.8)	180 (8.3)
Not intact	1274	100 (7.8)	34 (2.7)	95 (7.5)	107 (8.4)
Unknown	313	7 (2.2)	9 (2.9)	13 (4.2)	45 (14.4)
AKC breed group					
Herding	391	42 (10.7)	14 (3.6)	37 (9.5)	40 (10.2)
Hound	503	54 (10.7)	14 (2.8)	91 (18.1)	78 (15.5)
Non-sporting	159	18 (11.3)	3 (1.9)	9 (5.7)	9 (5.7)
Sporting	466	60 (12.9)	26 (5.6)	32 (6.9)	52 (11.2)
Terrier	1185	169 (14.3)	17 (1.4)	71 (6.0)	86 (7.3)
Toy	418	15 (3.6)	1 (0.2)	13 (3.1)	12 (2.9)
Working	268	21 (7.8)	6 (2.2)	19 (7.1)	31 (11.6)
Unknown	360	40 (11.1)	9 (2.5)	27 (7.5)	24 (6.7)
Region/State					
Northeast	740	12 (1.6)	42 (5.7)	34 (4.6)	143 (19.3)
Maine	189	2 (1.1)	22 (11.6)	4 (2.1)	44 (23.3)
Maryland	202	10 (5.0)	2 (1.0)	29 (14.4)	39 (19.3)
New Hampshire	37	0 (0)	2 (5.4)	1 (2.7)	5 (13.5)
New York	171	0 (0)	14 (8.2)	0 (0)	42 (24.6)
Pennsylvania	141	0 (0)	2 (1.4)	0 (0)	13 (9.2)
Midwest	974	52 (5.3)	19 (2.0)	48 (4.9)	66 (6.8)
Kansas	16	0 (0)	0 (0)	1 (6.3)	0 (0)
Minnesota	97	3 (3.1)	11 (11.3)	3 (3.1)	28 (28.9)
Missouri	335	36 (10.7)	2 (0.6)	37 (11.0)	1 (0.3)
North Dakota	2	0 (0)	0 (0)	0 (0)	0 (0)
Ohio	352	13 (3.7)	1 (0.3)	5 (1.4)	22 (6.3)
Wisconsin	172	0 (0)	5 (2.9)	2 (1.2)	15 (8.7)
Southeast	2036	355 (17.4)	29 (1.4)	217 (10.7)	123 (6.0)
Florida	372	59 (15.9)	0 (0)	18 (4.8)	3 (0.8)
Georgia	200	31(15.5)	0 (0)	9 (4.5)	1 (0.5)
Louisiana	177	59 (33.3)	1 (0.6)	4 (2.3)	0 (0)
Mississippi	116	35 (30.2)^a^	0 (0)	21 (18.1)	0 (0)
North Carolina	197	56 (28.4)	0 (0)	52 (26.4)	7 (3.6)
Oklahoma	200	26 (13.0)	0 (0)	18 (9.0)	0 (0)
Tennessee	382	75 (19.6)	4 (1.0)	13 (3.4)	1 (0.3)
Virginia	392	14 (3.6)	24 (6.1)	82 (20.9)	111 (28.3)

^a^Heartworm data from Mississippi is published (Donnett et al. [15])

### *Dirofilaria immitis* antigen seroprevalence

**Fig. 2:**
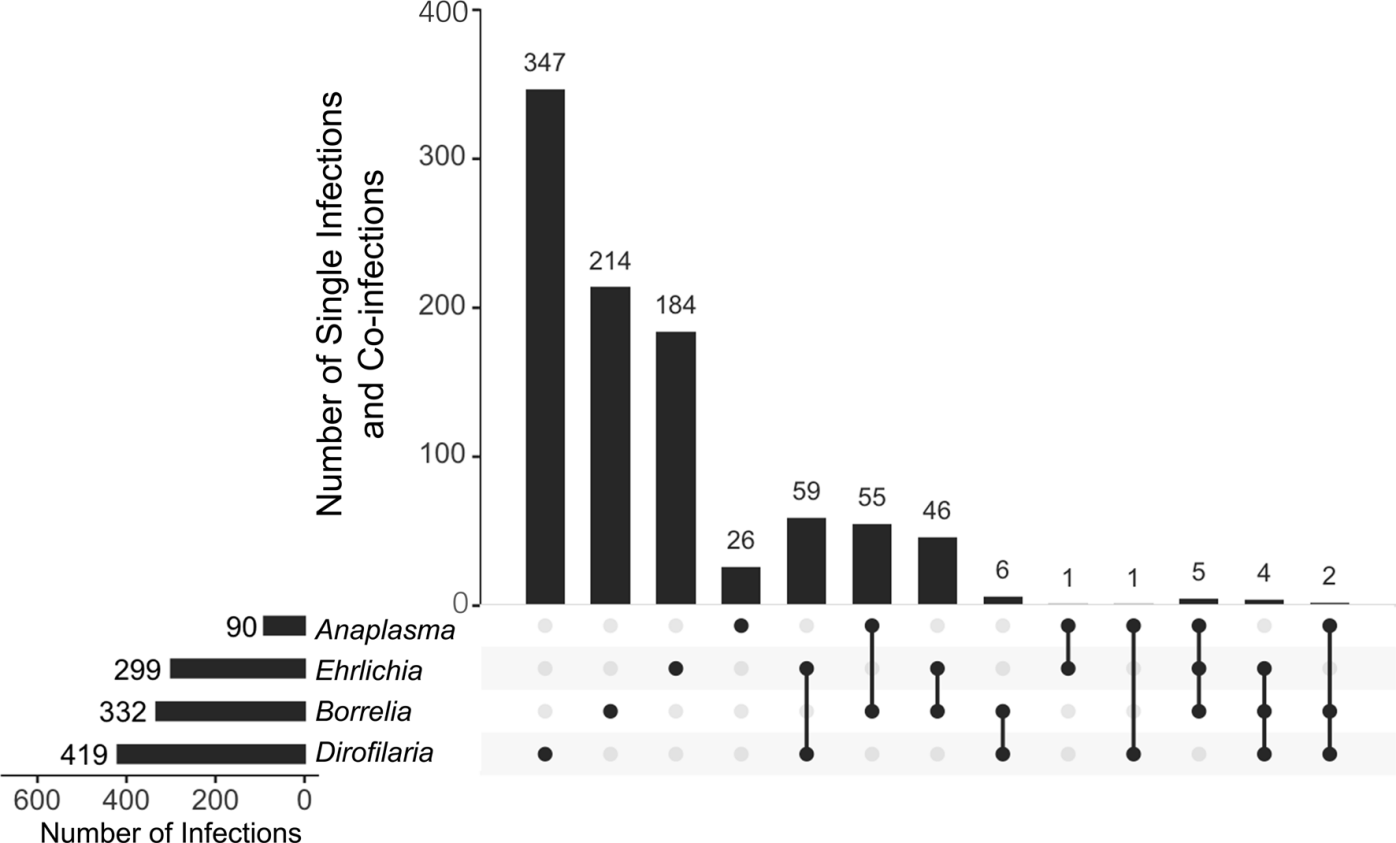
Bar plot of single seroprevalence data and co-infection data. The main bar plot represents the dogs that either had only one pathogen detected or had co-infections detected. The side bar plot represents the single seroprevalence data without the subtraction of the co-infections

A total of 419 (11.2%, *n* = 3750) dogs were positive for *D. immitis* antigens (Table 1, Fig. 2). Dogs < 1 year of age had a significantly lower seroprevalence and were 12.2 times less likely to have *D. immitis* antigens detected compared with dogs ≥ 1 year of age (Tables 1 and 2). A higher seroprevalence was noted for males than for females (Tables 1 and 2). Dogs that were intact were twice as likely to be positive as non-intact dogs (Tables 1 and 2). The seroprevalence of *D. immitis* antigens was lower in the toy breed group than in the herding, hound, non-sporting, sporting, and terrier breed groups (Tables 1, 2).

**Table 2:** Significant results of bivariable generalized linear model and pairwise comparison analysis of potential risk factors for seropositive status for vector-borne pathogens among 3750 dogs from shelters in 19 states in the Eastern United States

Pathogens and pairs of risk factors	OR	95% CI	*P* value
*Dirofilaria immitis*			
Age group			
≥ 1 year old vs. < 1 year old	12.2	5.4–27.4	< 0.0001
Sex			
Male–Female	1.2	1.0–1.5	0.0418
Intact status			
Intact–Non-intact	2.0	1.6–2.5	< 0.0001
Breed group			
Herding–Toy	3.2	1.7–5.9	0.0032
Hound–Toy	3.2	1.8–5.8	0.0020
Non-sporting–Toy	3.4	1.7–7.0	0.0120
Sporting–Toy	4.0	2.2–7.1	0.0001
Terrier–Toy	4.4	2.6–7.6	< 0.0001
Region			
Midwest–Northeast	3.4	1.8–6.4	0.0005
Southeast–Midwest	3.7	2.8–5.1	< 0.0001
Southeast–Northeast	12.7	7.1–22.7	< 0.0001
*Ehrlichia* spp.			
Age group			
≥ 1 year old vs. < 1 year old	3.7	2.1–6.5	< 0.0001
Breed group			
Herding–Toy	3.2	1.7–6.2	0.0073
Hound–Herding	2.1	1.4–3.2	0.0060
Hound–Non-sporting	3.6	1.8–7.4	0.0067
Hound–Sporting	3.0	1.9–4.6	< 0.0001
Hound–Terrier	3.5	2.5–4.8	< 0.0001
Hound–Toy	6.8	3.7–12.4	< 0.0001
Hound–Working	2.9	1.7–4.8	0.0013
Region			
Southeast–Midwest	2.3	1.7–3.2	< 0.0001
Southeast–Northeast	2.5	1.7–3.6	< 0.0001
*Anaplasma* spp.			
Breed group			
Sporting–Terrier	4.1	2.2–7.6	0.0002
Sporting–Toy	24.5	3.3–181.3	0.0288
Region			
Northeast–Midwest	3.1	1.8–5.3	0.0002
Northeast–Southeast	4.2	2.6–6.8	< 0.0001
*Borrelia burgdorferi*			
Age group			
≥ 1 year old vs. < 1 year old	3.5	2.0–5.9	< 0.0001
Breed group			
Herding–Toy	3.8	2.0–7.4	0.0014
Hound–Non-sporting	3.0	1.5–6.2	0.0388
Hound–Terrier	2.3	1.7–3.2	< 0.0001
Hound–Toy	6.1	3.3–11.5	< 0.0001
Sporting–Toy	4.2	2.2–8.0	0.0002
Terrier–Toy	2.6	1.4–4.9	0.0336
Working–Toy	4.4	2.2–8.8	0.0004
Region			
Northeast–Midwest	3.3	2.4–4.5	< 0.0001
Northeast–Southeast	3.8	2.9–4.9	< 0.0001

*OR* odds ratio, *CI* confidence interval

*Dirofilaria immitis* antigen detection was highest in dogs from the Southeast (17.4%, *n* = 355/2036) followed by dogs from the Midwest (5.3%, *n* = 52/974) and dogs from the Northeast (1.6%, *n* = 12/740) (Table 1, Fig. 1). Dogs from the Midwest were 3.4 times more likely to have *D. immitis* antigen detection than dogs from the Northeast (Table 2). Dogs in the Southeast were 3.7 times more likely to have *D. immitis* antigen detection than dogs from the Midwest and 12.7 times more likely than dogs from the Northeast (Table 2). An analysis of state vs. state comparisons is provided in Additional file 1: Text S1 and Table S1.

### *Ehrlichia* spp. antibody seroprevalence

*Ehrlichia* spp. antibodies were detected in 8.0% (*n* = 299/3750) of the dogs (Table 1, Fig. 2). Analyzed risk factors in relation to detection of *Ehrlichia* spp. antibodies of significance were age, breed group, and location (Table 2). Dogs ≥ 1 year of age were 3.7 times more likely to have *Ehrlichia* spp. antibody seroprevalence than dogs < 1 year of age (Table 2). The hound breed group had the highest seroprevalence of *Ehrlichia* spp. antibodies (Table 1). The hound group was at an increased risk of *Ehrlichia* spp. antibody detection compared to the other analyzed breed groups (Table 2). The herding breed group was found to be 3.2 times more likely to have *Ehrlichia* spp. antibody detected than the toy breed group (Table 2).

The highest seroprevalence of *Ehrlichia* spp. antibodies was documented in dogs from the Southeast (10.7%, *n* = 217/2036) (Table 1, Fig. 1). The lowest seroprevalence was documented in dogs from the Northeast (4.6%, *n* = 34/740) and dogs from the Midwest had a seroprevalence of 4.9% (*n* = 48/974) (Table 1). Dogs from the Southeast had a statistically higher risk of *Ehrlichia* spp. antibody seroprevalence (Table 4). Dogs from the Southeast were 2.5 times more likely to have *Ehrlichia* spp. antibody seroprevalence than dogs from the Northeast and 2.3 times more likely than dogs from the Midwest (Table 2). An analysis of state vs. state comparisons is provided in Additional file 1: Text S2 and Table S2.

### *Anaplasma* spp. antibody seroprevalence

*Anaplasma* spp. antibodies were detected in 2.4% (*n* = 90/3750) of the dogs (Table 1, Fig. 2). Risk factors that were significant in relation to *Anaplasma* spp. antibody seroprevalence included breed group and location (Table 2). The sporting breed group had the highest seroprevalence of *Anaplasma* spp. antibodies of 5.6% (*n* = 26/466) (Table 1). The sporting breed group was at an increased risk of *Anaplasma* spp. antibody seroprevalence compared to the terrier and toy breed groups (Table 2). The sporting breed group was 4.1 times more likely to have *Anaplasma* spp. antibodies detected than the terrier breed group (Table 2). The sporting breed group was 24.5 times more likely to have *Anaplasma* spp. antibodies detected than the toy breed group (Table 2).

The highest seroprevalence of *Anaplasma* spp. antibodies was documented in dogs from the Northeast (5.7%, *n* = 42/740) (Table 1, Fig. 1) followed by dogs from the Midwest (2.0%, *n* = 19/974) and dogs from the Southeast (1.4%, *n* = 29/2036) (Table 1, Fig. 1). Dogs from the Northeast were 4.2 times and 3.1 times more likely to have *Anaplasma* spp. antibodies than dogs from the Southeast and Midwest, respectively (Table 2). An analysis of state versus state comparisons is provided in Additional file 1: Text S3 and Table S3.

### *Borrelia burgdorferi* antibody seroprevalence

*Borrelia burgdorferi* antibodies were detected in 8.9% (*n* = 332/3750) of dogs (Table 1, Fig. 2). Risk factors found to be significantly related to detection of *B. burgdorferi* antibodies were age, breed group, and location (Table 2). Dogs ≥ 1 year of age were 3.5 times more likely to have *B. burgdorferi* antibodies than dogs < 1 year of age (Tables 1 and 2). The hound breed group had the highest seroprevalence of *B. burgdorferi* antibodies (15.5%, *n* = 78/503) (Table 1). The herding, hound, sporting, terrier, and working breed groups had significantly higher seroprevalence of *B. burgdorferi* compared to the other analyzed breed groups (Table 2). Additionally, the toy breed group (2.9%, *n* = 12/418) had a decreased risk of *B. burgdorferi* antibody detection (Table 2).

The highest *B. burgdorferi* antibody seroprevalence was documented in dogs from the Northeast (19.3%, *n* = 143/740) followed by dogs from the Midwest (6.8%, *n* = 66/974) and dogs from the Southeast (6.0%, *n* = 123/2036) (Table 1, Fig. 1). Dogs from the Northeast had an increased risk of *B. burgdorferi* antibody detection which was 3.3 times more likely than dogs from the Midwest and 3.8 times more likely than dogs from the Southeast (Table 2, Fig. 1). An analysis of state versus state comparisons is provided in Additional file 1: Text S4 and Table S4.

### Co-infections

**Fig. 3:**
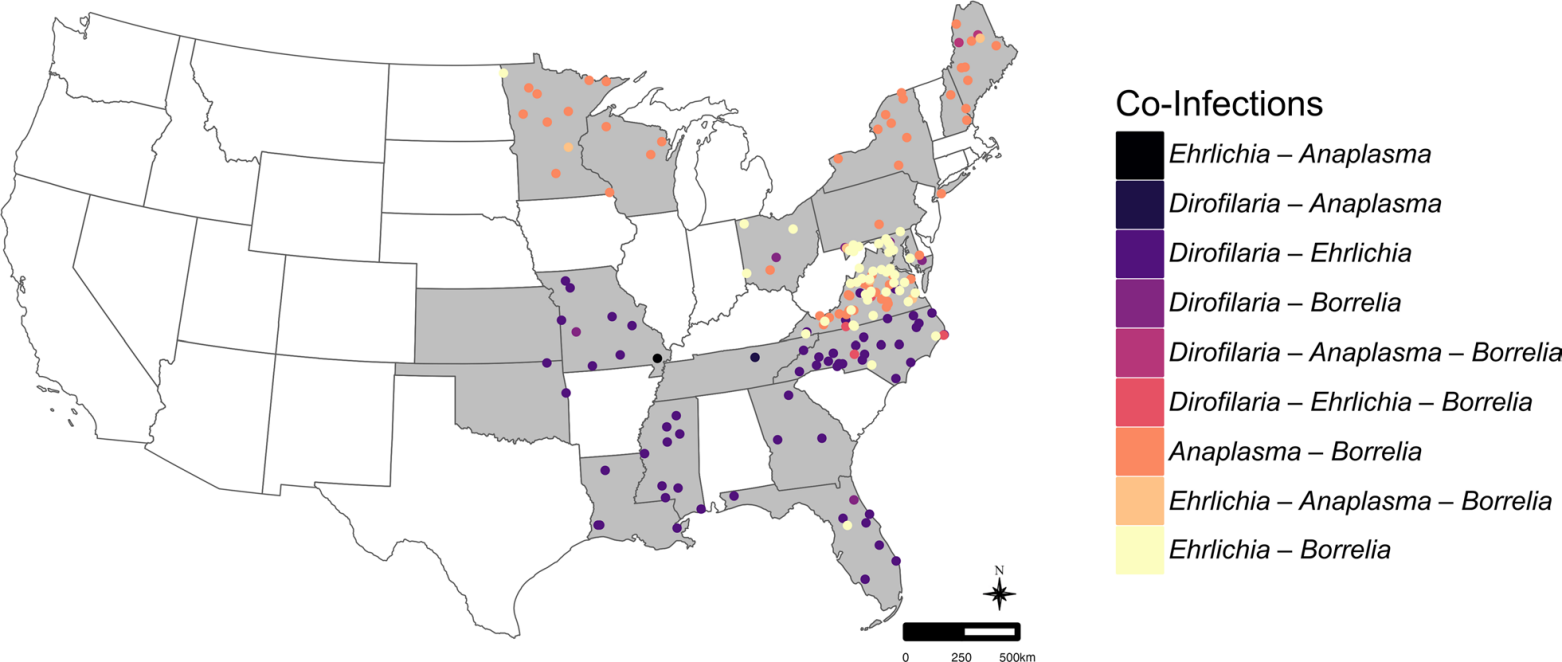
Map of co-infections included in the study. Each co-infection combination is represented with a unique color and the included states are shaded gray. The points are representative of a single co-infection and are randomly placed within the state of origin. The map was created in R statistical software (R Core Team)

There were nine different co-infection combinations observed in 179 dogs (Table 3, Fig. 2). The three most prevalent co-infections were *B. burgdorferi* + *Anaplasma* spp. (1.47%, *n* = 55/3750), *B. burgdorferi* + *Ehrlichia* spp. (1.23%, *n* = 46/3750), and *D. immitis* + *Ehrlichia* spp. (1.57%, *n* = 59/3750) (Tables 3, 4, Figs. 2, 3).

**Table 3:** Co-infections among 3750 dogs from shelters in 19 states in the Eastern United States

Pathogens				No. of co-infections	Location	Prevalence (%)
*Dirofilaria immitis*	*Borrelia burgdorferi*		*Ehrlichia* spp.	4	NC, VA	0.11
*D. immitis*	*B. burgdorferi*		*Anaplasma* spp.	2	ME	0.05
*B. burgdorferi*	*Anaplasma* spp.		*Ehrlichia* spp.	5	ME, MD, MN, VA	0.13
*D. immitis*	*Ehrlichia* spp.			59	FL, GA, LA, MS, MO, NC, OK, TN, VA	1.57
*D. immitis*	*Anaplasma* spp.			1	TN	0.03
*D. immitis*	*B. burgdorferi*			6	FL, MD, MO, OH	0.16
*B. burgdorferi*	*Ehrlichia* spp.			46	FL, MD, MN, NC, OH, VA	1.23
*B. burgdorferi*	*Anaplasma* spp.			55	MD, ME, MN, NH, NY, OH, PA, VA, WI	1.47
*Anaplasma* spp.	*Ehrlichia* spp*.*			1	MO	0.03
Total co-infections				179		4.77

**Table 4:** Demographic data and seroprevalence of infection with the three most prevalent co-infection combinations (*Borrelia burgdorferi* + *Anaplasma* spp., *B. burgdorferi* + *Ehrlichia* spp., and *Dirofilaria immitis* + *Ehrlichia* spp.) pathogens among 3750 dogs from shelters in 19 states in the Eastern United States

	*n*	*Dirofilaria immitis* + *Ehrlichia* spp. (%)	*Borrelia burgdorferi* + *Ehrlichia* spp. (%)	*Borrelia burgdorferi* + *Anaplasma* spp. (%)
Age group				
< 1 year old	492	1 (0.20)	1 (0.20)	4 (0.81)
≥ 1 year old	3100	58 (1.87)	44 (1.42)	47 (1.52)
Unknown	158	0(0)	1 (0.63)	4 (2.53)
Sex				
Female	1938	32 (1.65)	22 (1.14)	25 (1.29)
Male	1686	27 (1.60)	23 (1.36)	27 (1.60)
Unknown	126	0 (0)	1 (0.79)	3 (2.38)
Intact status				
Intact	2163	46 (2.13)	28 (1.29)	34 (1.57)
Non-intact	1274	13 (1.02)	13 (1.02)	16 (1.26)
Unknown	313	0 (0)	5 (1.60)	5 (1.60)
AKC breed group				
Herding	391	6 (1.53)	4 (1.02)	10 (2.56)
Hound	503	12 (2.39)	22 (4.37)	9 (1.79)
Non-Sporting	159	3 (1.89)	1 (0.63)	0 (0)
Sporting	466	9 (1.93)	3 (0.64)	16 (3.43)
Terrier	1185	16 (1.35)	13 (1.10)	11 (0.93)
Toy	418	1 (0.24)	1 (0.24)	0 (0)
Working	268	3 (1.12)	1 (0.37)	2 (0.75)
Unknown	360	9 (2.50)	1 (0.28)	7 (1.94)
Region				
Northeast	740	0 (0)	13 (1.76)	20 (2.70)
Maine	189	0 (0)	0 (0)	7 (3.70)
Maryland	202	0 (0)	13 (6.44)	1 (0.50)
New Hampshire	37	0 (0)	0 (0)	2 (5.41)
New York	171	0 (0)	0 (0)	9 (5.26)
Pennsylvania	141	0 (0)	0 (0)	1 (0.71)
Midwest	974	7 (0.72)	4 (0.41)	13 (1.33)
Kansas	16	0 (0)	0 (0)	0 (0)
Minnesota	97	0 (0)	1 (1.03)	9 (9.28)
Missouri	335	7 (2.09)	0 (0)	0 (0)
North Dakota	2	0 (0)	0 (0)	0 (0)
Ohio	352	0 (0)	3 (0.85)	1 (0.28)
Wisconsin	172	0 (0)	0 (0)	3 (1.74)
Southeast	2036	52 (2.55)	29 (1.42)	22 (1.08)
Florida	372	7 (1.88)	1 (0.27)	0 (0)
Georgia	200	3 (1.50)	0 (0)	0 (0)
Louisiana	177	4 (2.26)	0 (0)	0 (0)
Mississippi	116	9 (7.76)	0 (0)	0 (0)
North Carolina	197	22 (11.17)	2 (1.02)	0 (0)
Oklahoma	200	2 (1.00)	0 (0)	0 (0)
Tennessee	382	1 (0.26)	0 (0)	0 (0)
Virginia	392	4 (1.02)	26 (6.63)	22 (5.61)

*n* total number of samples

For all three of these co-infection pairs, location was a significant risk factor (Table 5). The *B. burgdorferi* + *Anaplasma* spp. pair was more likely to be detected in the Northeast than in the Southeast (Tables 4 and 5, Fig. 3). The co-infection combination between *B. burgdorferi* + *Ehrlichia* spp. was mostly observed in the Northeast and was significantly more likely to be detected in the Northeast than in the Midwest, although all of the Northeast positives were from Maryland (Table 5, Fig. 3). The co-infection pair of *D. immitis* + *Ehrlichia* spp. was significantly more likely to occur in the Southeast than the Midwest, and no cases were noted in the Northeast (Tables 4 and 5, Fig. 3).

**Table 5:** Significant results of the binomial generalized linear model and pairwise comparison analysis of potential risk factors for positive status for the three most common co-infections noted in this study among 3750 dogs from shelters in 19 states in the Eastern United States

Pairs and risk factors	OR	95% CI	*P* value
*Dirofilaria immitis* + *Ehrlichia* spp.			
Age group			
≥ 1 year old/< 1 year old	9.3	1.3–67.3	0.0269
Intact status			
Intact–Non-intact	2.1	1.1–3.9	0.0185
Region			
Southeast–Midwest	3.6	1.2–11.1	0.0042
*Borrelia burgdorferi* + *Ehrlichia* spp.			
Breed group			
Hound–Sporting	7.0	2.1–23.6	0.0272
Hound–Terrier	4.1	2.1–8.2	0.0013
Region			
Northeast–Midwest	4.4	1.4–13.5	0.0275
*Borrelia burgdorferi* + *Anaplasma* spp.			
Breed group			
Sporting–Terrier	3.8	1.8–8.3	0.0131
Region			
Northeast–Southeast	2.6	1.4–4.7	0.0072

*OR* odds ratio, *CI* confidence interval

There were also significant associations for breed group for co-infections *B. burgdorferi* + *Anaplasma* spp. and *B. burgdorferi* + *Ehrlichia* spp. (Table 5). The sporting breed group was 3.8 times more likely to have co-infections with *B. burgdorferi* + *Anaplasma* spp. than the terrier breed group (Table 5). The hound breed group was 7.0 times more likely to have co-infections with *B. burgdorferi* + *Ehrlichia* spp. than the sporting breed group and 4.1 times more likely than the terrier breed group (Table 5).

Additional risk factors for co-infections with *D. immitis* + *Ehrlichia* spp. were age group and intact status (Table 5). Dogs that were ≥ 1 year of age were 9.3 times more likely to have a co-infection with *D. immitis* + *Ehrlichia* spp. than dogs < 1 year of age (Table 5). Dogs that were intact were 2.1 times more likely to have co-infections with *D. immitis* + *Ehrlichia* spp. than dogs that were not intact (Table 5).

## Discussion

In the present study, we investigated the seroprevalence of VBP (*D. immitis, Ehrlichia* spp., *Anaplasma* spp., and *B. burgdorferi*) in shelter dogs in the Eastern United States from 2016 to 2020. We found regional variation in seroprevalence of all pathogens and several risk factors (age, sex, breed group, and intact status) were associated with infection. We also observed several different co-infection combinations with *B. burgdorferi* + *Anaplasma* spp., *B. burgdorferi* + *Ehrlichia* spp., and *D. immitis* + *Ehrlichia* spp. being the most frequently detected. This study provides contemporary data on the seroprevalence of these pathogens in a group of dogs that are expected to have limited veterinary care or preventative use. High seroprevalence and detection outside of known endemic regions highlight the need for continued monitoring.

Knowledge regarding the accurate distribution of pathogens is critically important for veterinarians and clients to gauge the risk of disease in dogs and other possible hosts. In general, the geographical distributions for the VBP were consistent with the known geographical ranges reported in previous studies for both the pathogens and vectors [1, 4, 5, 9, 11, 12, 15, 16, 22, 28]. However, there were some notable findings. We detected a low seroprevalence (1.1%, *n* = 2/189) of *D. immitis* in Maine which historically has few heartworm detections (< 0.5%) [11]. Detection of *D. immitis* outside the known endemic range is often assumed to be related to travel or translocated dogs from heartworm-endemic regions. However, the inclusion criteria of this study should have excluded most translocated dogs, and our findings are supported by increasing heartworm prevalence trends in the far Northeast and other regions (e.g., Colorado) [11, 21, 29, 30]. The potential for local transmission in non-endemic regions highlights the need for heartworm preventative use. In addition, we noted a higher seroprevalence of *B. burgdorferi* infection in dogs from Virginia compared to past studies using similar methods [11], which corresponds with reported changes in the distribution of this pathogen in dogs and people and its vector in Virginia [8, 31–34]. In addition, a low number of *B. burgdorferi*-positive dogs were detected in states where Lyme disease risk is low (e.g., Missouri, Florida, and Georgia). Our inclusion criteria should have excluded translocated dogs, but we do not know whether all shelters were 100% compliant (although few dogs from the Northeast/Upper Midwest where Lyme disease is more common are moved south). Additional studies are needed to investigate the possible transmission of *B. burgdorferi* in these areas. Similarly, a few states had notable detections of *Ehrlichia* (e.g., Maine, New Hampshire, Minnesota, and Wisconsin). Although the species involved is unknown, these detections may be due to the well-documented northern expansion of *A. americanum* in the Northeast or *E. muris eauclairensis* in Minnesota and Wisconsin [24, 25, 35]. Continued studies on the distribution of VBP are warranted, as changes in the distribution and density of vectors and their associated pathogens have been noted in recent years, which may be related to several factors such as climate or habitat changes [5, 8, 20–23, 36]. Additionally, novel vectors (e.g., Asian longhorned tick, *Haemaphysalis longicornis*) have been introduced into the United States, and this tick may alter the native pathogen transmission dynamics [37–41].

Consistent with previous studies, dogs that were ≥ 1 year of age had an increased risk of being positive [7, 15]. Tick-borne pathogen infection was assessed with the detection of antibodies which may be persistent; older dogs have an increased time at risk of exposure, increasing the likelihood of infection to the vector and pathogen. Heartworm infections also typically occur in older dogs as this parasite has a long life cycle and older dogs have increased time for mosquito exposure [42]. We also found that intact dogs were more likely to be infected with *D. immitis* compared with non-intact dogs, who may be more likely to have previous access to veterinary care or preventative medications. Other studies have also noted that intact dogs had higher rates of VBP infection [43, 44] and tick exposure [45].

Interestingly, we found that breed group was associated with changes in infection risk. Our findings indicate that the toy breed group specifically had a lower risk of infection for all pathogens, which is consistent with a previous study on ticks [45]. The decreased risk of infection for the toy breed group may be due to the popularity of these dogs in urban settings or that smaller dogs likely spend less time outdoors. Similarly, larger breed groups (e.g., hound, herding, sporting) had higher seroprevalence of infection, which may be related to more time spent outdoors. There may also be spatial differences in breed distribution because of different trends in owner popularity and dog utility. For example, both *D. immitis* and *Ehrlichia* were more common in large breed groups that are popular outdoor dogs in Southern states. Although this study focused on VBP, similar results (increased risk of parasitic infections for large breed groups) have been noted in other parasite systems (e.g., *Dracunculus*) [46, 47].

In general, the seroprevalence of pathogens included in this study was comparable to previous shelter-based studies conducted in the same regions using similar methods (e.g., 16.0% *D. immitis* seroprevalence in Texas) [7]. However, our seroprevalence rates for *D. immitis*, *B. burgdorferi*, and *Ehrlichia* spp. were higher than those in other studies primarily conducted on owned dogs and public data available on the Companion Animal Parasite Council (CAPC) website [11, 28]. Our observed higher seroprevalence rates most likely were because our sample collection was from shelter dogs, and > 50% of the dogs that enter a shelter are considered strays [13]. These dogs are expected to have decreased access to veterinary care and therefore preventative medications. Further analysis of this association is reported separately [48].

Dogs are not exposed to vectors or pathogens in isolation, and most geographical regions have multiple vectors and pathogens co-circulating. Many large serosurvey studies on dogs and pathogen infection are unable to examine co-infections because testing data are not associated with individual cases [11, 21]. However, prospective studies, such as this one, provide opportunities to examine patterns of co-infections [7]. Although we detected nine co-infection groups, including 11 dogs with infection with three pathogens, the most common pairs were *B. burgdorferi* + *Anaplasma* spp., *B. burgdorferi* + *Ehrlichia* spp., and *D. immitis* + *Ehrlichia* spp. The most likely explanation for the increased frequency between these three combinations is because of the pathogens' shared ranges and vectors. *Anaplasma phagocytophilum* and *B. burgdorferi* have overlapping ranges in the Northeast, which was the region of highest seroprevalence documented in our study, and share the same vector (*I. scapularis)* [9, 49]. Co-infections between *B. burgdorferi* and *Ehrlichia* spp., and *D. immitis* and *Ehrlichia* spp. were most likely more common due to overlapping ranges. The *Ehrlichia* spp. most commonly detected in our study is likely *E. canis*, due to its documented prevalence in the South; however, other *Ehrlichia* spp. are possible [9].

## Conclusions

In this study, we analyzed seroprevalence data for VBP in 3750 dogs sampled in shelters from 97 counties in 19 states in the Eastern United States. In general, we found *D. immitis* and *Ehrlichia* spp. seroprevalence to be highest in the Southeast and *Anaplasma* spp. and *B. burgdorferi* seroprevalence to be highest in the Northeast, which is consistent with previous studies and expected vector ranges. Co-infections that were most common were between *B. burgdorferi* + *Anaplasma* spp., *B. burgdorferi* + *Ehrlichia* spp., and *D. immitis* + *Ehrlichia* spp. We found decreased risk of infection in dogs that were less than 1 year of age, in the toy breed group, and in dogs that had been spayed or neutered. In general, we found increased risk of infection in dogs that were more than 1 year of age and in the hound breed group. However, some pathogens were detected outside their typical range, so these data support previous studies that show an expanding range for these pathogens and/or vector species, highlighting the need for continued surveillance and assessment of risk factors in both owned and unowned dogs.

## Supplementary Information

**Additional file 1.** State-level statistical analysis of the seroprevalence of the four vector-borne pathogens in domestic dogs between each pair of states. 

## Data Availability

Aggregated data from shelters are provided in the manuscript and are available from the corresponding author on reasonable request.
