# Supplementary material for: Spatial and risk factor analyses of vector-borne pathogens among shelter dogs in the Eastern United States
Source: Parasit Vectors. 2023 Jun 10;16:197. doi: 10.1186/s13071-023-05813-1 (PMC10257847; doi:10.1186/s13071-023-05813-1)
Supplement: Supplementary file 1 — Additional file 1. State-level statistical analysis of the seroprevalence of the four vector-borne pathogens in domestic dogs between each pair of states. [file 13071_2023_5813_MOESM1_ESM.docx]

**Additional File 1**

**State-level statistical analysis of the seroprevalence of vector-borne pathogens in domestic dogs**

In addition to the Region-level analysis provided in the main text of the manuscript, we also analyzed each state separately for significant differences in seroprevalence for each pathogen between each pair of states.

***Dirofilaria immitis* Antigen Serorevalence**

The state with the observed highest seroprevalence of *D. immitis* was Louisiana 33.3% (n = 59/177) and no dogs from New Hampshire, New York, Pennsylvania, Kansas, North Dakota, and Wisconsin were positive for *D. immitis* antigens (Table 1). States that were significantly more likely to have dogs positive for *D. immitis* antigens were Florida (15.9%, n = 59/372), Georgia (15.5%, n = 31/200), Louisiana, Mississippi (30.2%, n = 35/116), Missouri (10.7%, n = 36/335), North Carolina (28.4%, n = 56/197), Oklahoma (13.0%, n = 26/200), and Tennessee (19.6%, n = 75/382) (Table 1 and S1).

Table S1 Significant results of bivariable generalized linear model and pairwise comparison analysis of different states for positive status of *Dirofilaria immitis* among 3,750 dogs from shelters in 19 states in the eastern United Stat*e*s

| **Pairs of States** | **OR** | **95% CI** | ***P* value** |
| --- | --- | --- | --- |
| State |  |  |  |
| Florida – Maine | 17.6 | 4.3 – 73.0 | 0.0104 |
| Florida – Maryland | 3.6 | 1.8 – 7.1 | 0.0397 |
| Florida – Ohio | 4.9 | 2.6 – 9.1 | 0.0001 |
| Florida – Virginia | 5.1 | 2.8 – 9.3 | <0.0001 |
| Georgia – Maine | 17.9 | 4.2 – 75.9 | 0.0124 |
| Georgia – Ohio | 5.0 | 2.5 – 9.8 | 0.0005 |
| Georgia – Virginia | 5.2 | 2.7 – 10.0 | 0.0002 |
| Louisiana – Florida | 2.7 | 1.7 – 4.0 | 0.0008 |
| Louisiana – Georgia | 2.6 | 1.6 – 4.3 | 0.0187 |
| Louisiana – Maine | 46.7 | 11.2 – 195.1 | <0.0001 |
| Louisiana – Maryland | 9.4 | 4.6 – 19.2 | <0.0001 |
| Louisiana – Minnesota | 15.7 | 4.8 – 51.6 | 0.0009 |
| Louisiana – Missouri | 4.2 | 2.6 – 6.6 | <0.0001 |
| Louisiana – Ohio | 13.0 | 6.9 – 24.6 | <0.0001 |
| Louisiana – Oklahoma | 3.3 | 2.0 – 5.6 | 0.0007 |
| Louisiana – Virginia | 13.5 | 7.3 – 25.0 | <0.0001 |
| Mississippi – Maine | 40.4 | 9.5 – 172.0 | 0.0001 |
| Mississippi – Maryland | 8.2 | 3.9 – 17.3 | <0.0001 |
| Mississippi – Minnesota | 13.5 | 4.0 – 45.6 | 0.0039 |
| Mississippi – Missouri | 3.6 | 2.1 – 6.1 | 0.0003 |
| Mississippi – Ohio | 11.3 | 5.7 – 22.3 | <0.0001 |
| Mississippi – Oklahoma | 2.9 | 1.6 – 5.1 | 0.0332 |
| Mississippi – Virginia | 11.7 | 6.0 – 22.7 | <0.0001 |
| Missouri – Virginia | 3.3 | 1.7 – 6.1 | 0.0338 |
| North Carolina – Florida | 2.1 | 1.4 – 3.2 | 0.0416 |
| North Carolina – Maine | 37.7 | 9.0 – 157.0 | 0.0001 |
| North Carolina – Maryland | 7.6 | 3.8 – 15.4 | <0.0001 |
| North Carolina – Minnesota | 12.6 | 3.8 – 41.5 | 0.0044 |
| North Carolina – Missouri | 3.3 | 2.1 – 5.3 | 0.0001 |
| North Carolina – Ohio | 10.5 | 5.6 – 19.8 | <0.0001 |
| North Carolina – Oklahoma | 2.7 | 1.6 – 4.5 | 0.0211 |
| North Carolina – Virginia | 10.9 | 5.9 – 20.2 | <0.0001 |
| Oklahoma – Maine | 14.0 | 3.3 – 59.7 | 0.0438 |
| Oklahoma – Ohio | 3.9 | 2.0 – 7.8 | 0.0150 |
| Oklahoma – Virginia | 4.0 | 2.1 – 7.9 | 0.0071 |
| Tennessee – Maine | 22.8 | 5.5 – 94.0 | 0.0022 |
| Tennessee – Maryland | 4.6 | 2.3 – 9.2 | 0.0018 |
| Tennessee – Ohio | 6.4 | 3.5 – 11.7 | <0.0001 |

OR = odds ratio; CI = confidence interval

***Ehrlichia spp.* Antibody Prevalence**

The state with the observed highest detection prevalence of *Ehrlichia* spp. antibodies was North Carolina (26.4%, n = 52/197) (Table 1). None of the dogs from New York, Pennsylvania, and North Dakota were positive for *Ehrlichia* spp. antibodies (Table 1). States that were significantly more likely to have detection of *Ehrlichia* spp. antibodies were Maryland (14.4%, n = 29/202), Mississippi (18.1%, n = 21/116), Missouri (11.0%, n = 37/335), North Carolina, Oklahoma (9.0%, n = 18/200), and Virginia (20.9%, n = 82/392) (Table 1 and S2).

Table S2 Significant results of bivariable generalized linear model and pairwise comparison analysis of different states for positive status of *Ehrlichia* spp. among 3,750 dogs from shelters in 19 states in the eastern United States

| **Pairs of States** | **OR** | **95% CI** | ***P* value** |
| --- | --- | --- | --- |
| State |  |  |  |
| Maryland – Florida | 3.4 | 1.8 – 6.2 | 0.0155 |
| Maryland – Louisiana | 7.4 | 2.5 – 21.4 | 0.0297 |
| Maryland – Maine | 7.9 | 2.7 – 22.9 | 0.0189 |
| Maryland – Ohio | 11.8 | 4.5 – 31.1 | 0.0001 |
| Maryland – Tennessee | 4.8 | 2.5 – 9.5 | 0.0008 |
| Maryland – Wisconsin | 14.1 | 3.3 – 59.9 | 0.0410 |
| Mississippi – Florida | 4.3 | 2.2 – 8.5 | 0.0025 |
| Mississippi – Georgia | 4.5 | 2.0 – 10.3 | 0.0369 |
| Mississippi – Louisiana | 9.6 | 3.2 – 28.7 | 0.0078 |
| Mississippi – Maine | 10.2 | 3.4 – 30.6 | 0.0048 |
| Mississippi – Ohio | 15.3 | 5.6 – 41.8 | <0.0001 |
| Mississippi – Tennessee | 6.3 | 3.0 – 13.0 | 0.0001 |
| Mississippi – Wisconsin | 18.2 | 4.2 – 79.5 | 0.0148 |
| Missouri – Ohio | 8.6 | 3.3 – 22.2 | 0.0013 |
| Missouri – Tennessee | 3.5 | 1.8 – 6.8 | 0.0190 |
| North Carolina – Florida | 7.2 | 4.0 – 12.7 | <0.0001 |
| North Carolina – Georgia | 7.4 | 3.5 – 15.6 | <0.0001 |
| North Carolina – Louisiana | 15.7 | 5.6 – 44.5 | <0.0001 |
| North Carolina – Maine | 16.8 | 5.9 – 47.6 | <0.0001 |
| North Carolina – Minnesota | 11.4 | 3.5 – 37.5 | 0.0088 |
| North Carolina – Missouri | 2.9 | 1.8 – 4.7 | 0.0010 |
| North Carolina – Ohio | 25.2 | 9.9 – 64.5 | <0.0001 |
| North Carolina – Oklahoma | 3.7 | 2.1 – 6.6 | 0.0016 |
| North Carolina – Tennessee | 10.3 | 5.5 – 19.5 | <0.0001 |
| North Carolina – Wisconsin | 30.0 | 7.2 – 125.5 | 0.0005 |
| Oklahoma – Ohio | 6.9 | 2.5 – 18.8 | 0.0226 |
| Virginia – Florida | 5.2 | 3.1 – 8.9 | <0.0001 |
| Virginia – Georgia | 5.4 | 2.7 – 11.0 | 0.0005 |
| Virginia – Louisiana | 11.4 | 4.1 – 31.8 | 0.0005 |
| Virginia – Maine | 12.2 | 4.4 – 33.9 | 0.0002 |
| Virginia – Minnesota | 8.3 | 2.6 – 26.8 | 0.0483 |
| Virginia – Missouri | 2.1 | 1.4 – 3.2 | 0.0473 |
| Virginia – Ohio | 18.4 | 7.3 – 45.8 | <0.0001 |
| Virginia – Oklahoma | 2.7 | 1.6 – 4.6 | 0.0438 |
| Virginia – Tennessee | 7.5 | 4.1 – 13.7 | <0.0001 |
| Virginia – Wisconsin | 21.8 | 5.3 – 89.8 | 0.0029 |

OR = odds ratio; CI = confidence interval

***Anaplasma spp.* Antibody Serorevalence**

The state with the observed highest prevalence was Maine (11.6%, n = 22/189) (Table 1). States with no positive dogs included Kansas, North Dakota, Florida, Georgia, Mississippi, North Carolina, and Oklahoma (Table 1). Maine, Minnesota (11.3%, n = 11/97), and New York (8.2%, n = 14/171) statistically had higher risks of *Anaplasma* spp. detection than the other surveyed states (Table 1 and S3). Dogs sampled in Maine were 22.0 times more likely to have *Anaplasma* spp. antibodies detected than dogs in Missouri (CI=5.1-94.5, *P* value=0.0049), 46.2 times more likely to have *Anaplasma* spp. antibodies detected than dogs in Ohio (CI=6.1-348.1, *P* value=0.0239), and 12.4 times more likely to have *Anaplasma* spp. antibodies detected than dogs in Tennessee (CI=4.2-36.7, *P* value=0.0008) (Table S3). Dogs sampled in Minnesota were 21.3 times more likely to have *Anaplasma* spp. antibodies detected than dogs in Missouri (CI=4.6-97.8, *P* value=0.0115), 44.9 times more likely to have *Anaplasma* spp. antibodies detected than dogs in Ohio (CI=5.7-351.5, *P* value=0.0357), and 12.1 times more likely to have *Anaplasma* spp. antibodies detected than dogs in Tennessee (CI=3.8-38.9, *P* value=0.0042) (Table S3). Dogs sampled in New York were 15.4 times more likely to have *Anaplasma* spp. antibodies detected than dogs in Missouri (CI=3.5-68.7, *P* value=0.0393) and 8.8 times more likely to have *Anaplasma* spp. antibodies detected than dogs in Tennessee (CI=2.8-27.0, *P* value=0.0207) (Table S3).

Table S3 Significant results of bivariable generalized linear model and pairwise comparison analysis of different states for positive status of *Anaplasma* spp. among 3,750 dogs from shelters in 19 states in the eastern United States

| **Pairs of States** | **OR** | **95% CI** | ***P* value** |
| --- | --- | --- | --- |
| State |  |  |  |
| Maine – Missouri | 22.0 | 5.1 – 94.5 | 0.0049 |
| Maine – Ohio | 46.2 | 6.1 – 348.1 | 0.0239 |
| Maine – Tennessee | 12.4 | 4.2 – 36.7 | 0.0008 |
| Minnesota – Missouri | 21.3 | 4.6 – 97.8 | 0.0115 |
| Minnesota – Ohio | 44.9 | 5.7 – 351.5 | 0.0357 |
| Minnesota – Tennessee | 12.1 | 3.8 – 38.9 | 0.0042 |
| New York – Missouri | 15.4 | 3.5 – 68.7 | 0.0393 |
| New York – Tennessee | 8.8 | 2.8 – 27.0 | 0.0207 |

OR = odds ratio; CI = confidence interval

***Borrelia burgdorferi* Antibody Prevalence**

The state with the observed highest prevalence was Minnesota (28.9%, n = 28/97) (Table 1). States with positive dogs included Kansas, North Dakota, Louisiana, Mississippi, and Oklahoma (Table 1). All the states in the Northeastern region were significantly more likely to test positive for *B. burgdorferi* antibodies (Table S4). Other states that were significantly more likely to have higher *B. burgdorferi* prevalence compared to other states included Minnesota, Wisconsin, and Virginia (Table S4).

Table S4 Significant results of bivariable generalized linear model and pairwise comparison analysis of different states for positive status of *Borrelia burgdorferi* among 3,750 dogs from shelters in 19 states in the eastern United States

| **Pairs of States** | **OR** | **95% CI** | ***P* value** |
| --- | --- | --- | --- |
| State |  |  |  |
| Maryland – Florida | 30.0 | 9.1 – 98.5 | <0.0001 |
| Maryland – Georgia | 46.8 | 6.4 – 344.2 | 0.0205 |
| Maryland – Missouri | 81.4 | 11.1 – 597.5 | 0.0023 |
| Maryland – North Carolina | 6.5 | 2.9 – 15.0 | 0.0015 |
| Maryland – Ohio | 3.7 | 2.1 – 6.4 | 0.0008 |
| Maryland – Tennessee | 92.9 | 12.7 – 681.7 | 0.0013 |
| Maine – Florida | 37.3 | 11.4 – 122.2 | <0.0001 |
| Maine – Georgia | 58.3 | 7.9 – 427.7 | 0.0205 |
| Maine – Missouri | 101.4 | 13.8 – 742.5 | 0.0009 |
| Maine – North Carolina | 8.1 | 3.6 – 18.6 | 0.0001 |
| Maine – Ohio | 4.6 | 2.6 – 7.9 | <0.0001 |
| Maine – Tennessee | 115.6 | 15.8 – 847.0 | 0.0005 |
| Minnesota – Florida | 49.9 | 14.8 – 168.6 | <0.0001 |
| Minnesota – Georgia | 77.9 | 10.4 – 583.2 | 0.0033 |
| Minnesota – Missouri | 135.5 | 18.1 – 1012.482 | 0.0003 |
| Minnesota – North Carolina | 10.9 | 4.6 – 26.1 | <0.0001 |
| Minnesota – Ohio | 6.1 | 3.3 – 11.3 | <0.0001 |
| Minnesota – Pennsylvania | 4.0 | 1.9 – 8.2 | 0.0209 |
| Minnesota – Tennessee | 154.6 | 20.7 – 1155.0 | 0.0001 |
| Minnesota – Wisconsin | 4.1 | 2.1 – 8.2 | 0.0080 |
| New Hampshire – Florida | 19.2 | 4.4 – 84.1 | 0.0118 |
| New Hampshire – Missouri | 52.2 | 5.9 – 460.5 | 0.0435 |
| New Hampshire – Tennessee | 59.5 | 6.7 – 525.3 | 0.0290 |
| New York – Florida | 42.0 | 12.8 – 138.0 | <0.0001 |
| New York - Georgia | 65.6 | 8.9 – 482.2 | 0.0057 |
| New York – Missouri | 114.0 | 15.5 – 837.1 | 0.0005 |
| New York – North Carolina | 9.2 | 4.0 – 21.1 | <0.0001 |
| New York – Ohio | 5.1 | 2.9 – 8.9 | <0.0001 |
| New York – Pennsylvania | 3.4 | 1.7 – 6.6 | 0.0449 |
| New York – Tennessee | 130.1 | 17.7 – 954.9 | 0.0003 |
| New York - Wisconsin | 3.5 | 1.8 – 6.5 | 0.0170 |
| Pennsylvania - Florida | 12.5 | 3.5 – 44.6 | 0.0133 |
| Virginia – Florida | 48.6 | 15.3 – 154.4 | <0.0001 |
| Virginia – Georgia | 75.8 | 10.5 – 548.0 | 0.0027 |
| Virginia – North Carolina | 10.6 | 4.8 – 23.3 | <0.0001 |
| Virginia – Missouri | 131.9 | 18.3 – 951.4 | 0.0002 |
| Virginia – Ohio | 5.9 | 3.7 – 9.6 | <0.0001 |
| Virginia – Pennsylvania | 3.9 | 2.1 – 7.2 | 0.0020 |
| Virginia – Tennessee | 150.5 | 20.9 – 1085.4 | 0.0001 |
| Virginia – Wisconsin | 4.0 | 2.3 – 7.1 | 0.0004 |
| Wisconsin – Florida | 12.1 | 3.5 – 42.6 | 0.0129 |

OR = odds ratio; CI = confidence interval
